# Automated Anomaly Detection in Blast Furnace Shaft Static Pressure Using Adversarial Autoencoders and Mode Decomposition

**DOI:** 10.3390/s25113473

**Published:** 2025-05-31

**Authors:** Xiaodong Sun, Jie Zhu, Bing Tang, Zhaohui Jiang

**Affiliations:** 1 School of Automation, Central South University, Changsha 410083, China; xiaodong.sun@cisdi.com.cn; 2 CISDI Information Technology (Chongqing) Co., Ltd., Chongqing 401122, China; jie.zhu@cisdi.com.cn (J.Z.); bing.tang@cisdi.com.cn (B.T.)

**Keywords:** blast furnace monitoring, time-series anomaly detection, adversarial autoencoder, variational mode decomposition, unsupervised learning

## Abstract

Monitoring the blast furnace shaft static pressure is crucial for maintaining a stable ironmaking process. Traditional rule-based methods and manual inspections suffer from high labor costs and inconsistent standards. This article proposes a new unsupervised anomaly detection framework that combines adversarial autoencoder with variational mode decomposition (VMD). Firstly, using VMD combined with sample entropy calculation and clustering algorithm, the trend, period, and other components of multidimensional signals are extracted, and then these components are integrated into an improved adversarial training autoencoder to detect global and local anomalies. The proposed method has an accuracy of 0.95, a recall rate of 0.91, and an F1 score of 0.93. Which demonstrates the method effectively captures multi-scale anomalies including value bias, morphological changes, and sudden fluctuations, while providing analysts with interpretable anomaly detail diagnosis.

## 1. Introduction

Blast furnace operators need to determine the blast furnace production status and make appropriate operational adjustments based on time-series signals (such as temperature, pressure, etc.) collected by sensors installed at different positions of the furnace. The distribution of gas flow inside the blast furnace is a critical factor affecting stable and smooth operation. To more comprehensively understand the gas flow distribution state within the blast furnace, many steel plants have introduced shaft static pressure measurement signals. Shaft static pressure is measured by evenly installing pressure sensors around the cooling walls at specific layers of the blast furnace. Different abnormal patterns of shaft static pressure often indicate abnormal gas flow distribution inside the furnace. Currently, the primary method for monitoring abnormal states of shaft static pressure requires operators to observe signal curves in real time, which consumes significant human effort and is difficult to standardize due to differences in analytical logic among operators. Therefore, constructing an automated algorithm for blast furnace shaft static pressure anomaly detection has become necessary. This method can significantly reduce the workload of blast furnace operators, standardize evaluation logic, and improve the stability of production control.

In traditional industrial fields, such as blast furnace process control systems (PCS), the anomaly detection logic for key indicators mainly relies on explicit expression rules and corresponding thresholds [1,2]. The drawback of this method is its high requirement for threshold setting, typically requiring process experts to adjust numerous parameters. Additionally, changes in raw materials, operational systems, and other influencing factors cause corresponding changes in the data distribution of blast furnace conditions and their monitoring indicators. This necessitates continuous threshold adjustments to adapt to complex changes in furnace conditions, making traditional rule-based anomaly detection methods difficult to apply in actual production processes. To optimize this situation, subsequent academic research proposed a series supervised machine learning (SML) based parameter optimization methods to achieve automatic adaptive parameter adjustment or fuzzy logic control (FLC) [3,4], but these still require manual design of rule-based process feature extraction as a foundation.

Since the key monitoring indicators in the blast furnace production process are time-series signal data collected through multiple sensors, the anomaly detection of these key indicators can be abstracted as a multidimensional time-series anomaly detection algorithm task. In recent years, researchers have developed various methods to address this problem. The most commonly used techniques can be divided into two major categories: traditional statistical models and deep learning-based approaches. Among them, statistical model-based methods include: distance-based models using Dynamic Time Warping (DTW) [5]; time-series prediction models represented by ARIMA [6]; and clustering algorithms represented by DBSCAN [7]. These traditional statistical models primarily focus on single-variable time-series anomaly detection. However, for multidimensional time-series anomaly detection as encountered in this study, these methods often perform unsatisfactorily due to the curse of dimensionality.

Deep learning algorithms for time-series anomaly detection [8,9,10,11,12,13,14,15,16,17,18] can be broadly categorized into two types. One type involves establishing prediction models to calculate prediction errors, such as recurrent neural networks (RNN) and Long Short-Term Memory (LSTM) networks [19,20], which extend traditional statistical model-based time-series prediction methods into the domain of deep learning. Since anomaly detection in industrial applications often lacks large amounts of labeled negative samples to support prediction model training, most prediction error-based models compare predicted values with observed values. This requires predicting time-series over extended periods, where high accuracy in long-term prediction is a prerequisite for effective anomaly detection models—a problem that has long challenged the industrial sector. The other type uses encoder-decoder architectures to reconstruct observed values and calculate reconstruction errors, with common models including autoencoder neural network (AE) and Variational Autoencoders (VAE) [21,22,23,24]. However, VAE models often struggle to learn true posterior distributions, which affects the quality of reconstruction [25,26].

In response to the aforementioned technical challenges, the academic community has attempted to enhance the generative performance of Variational Autoencoders (VAEs) by incorporating the adversarial mechanism of Generative Adversarial Networks (GANs), leading to the development of the Adversarial Autoencoder (AAE) algorithmic framework [25,26]. Within the domain of anomaly detection, the existing literature primarily explores AAE algorithms through two distinct research trajectories: the first category focuses on adaptive designs of generators and discriminators [27], aiming to improve anomaly recognition accuracy by optimizing feature extraction efficacy during adversarial training; the second category endeavors to integrate AAE with conventional anomaly detection models such as Isolation Forest and Support Vector Data Description (SVDD) [28,29], thereby reinforcing detection robustness in specific scenarios through ensemble learning strategies. Existing research demonstrates that the performance optimization of anomaly detection models critically depends on domain-specific customized modeling. Throughout this process, the effectiveness of data feature representation emerges as a pivotal constraining factor. For industrial time-series signals, time-frequency analysis techniques including Fourier Transform and Wavelet Transform have been extensively employed as data preprocessing methods. Among these, Variational Mode Decomposition (VMD) has garnered significant attention due to its prominent advantages in processing non-stationary signals with strong interference. In the field of anomaly monitoring, current studies on VMD methodology predominantly manifest in two aspects: The first involves adaptive selection mechanism design for critical parameters such as modal quantities [30]; The second combines VMD with deep learning models like Convolutional Autoencoders (CAE) to achieve anomaly identification in multidimensional industrial signals (e.g., hydro-turbine unit monitoring data [31]. Notably, the majority of the existing literature integrating VMD with neural networks concentrates on time-series prediction tasks, such as the construction of crude oil price prediction models based on VMD and Bidirectional Long Short-Term Memory (BiLSTM) networks [32]. However, the data characteristics in the aforementioned research contexts exhibit substantial differences from the blast furnace shaft static pressure data investigated in this study. Specifically, existing methods typically employ post-processing strategies such as Intrinsic Mode Functions (IMFs) component screening or convolutional feature extraction. When applied to blast furnace static pressure signal analysis, these approaches confront two primary limitations: firstly, inadequate adaptability to multi-scale signal characteristics under complex blast furnace operating conditions; secondly, insufficient interpretability of the feature processing procedures. In light of this, the present study proposes an innovative analytical framework tailored to the data characteristics of blast furnace shaft static pressure, aiming to achieve automated detection and interpretation of anomalous signals within this specific context.

The shaft static pressure sensors are evenly installed on multiple layers of blast furnace stave coolers, collectively characterizing the three-dimensional pressure field inside the furnace [33]. Therefore, unlike traditional anomaly detection that only considers temporal anomalies in time-series signals, this study also needs to consider spatial anomalies such as changes in relative magnitudes between different signals. Process experts refer to this analysis as furnace profile analysis, which was previously conducted by manually observing changes in relative values between different sensor signals to determine whether the furnace profile had changed.

During temporal anomaly detection, operations such as burden charging and blast injection cause shaft static pressure data to exhibit superimposed multiple periodicities with variable cycle lengths. Traditional manual observation methods can filter out these interference patterns using prior knowledge or experience to focus on abnormal fluctuations outside normal periodic variations. However, a key challenge in this study is how models that minimize overall reconstruction error can overcome these interferences to detect truly meaningful anomalies.

Due to the difficulty in obtaining labeled samples for blast furnace shaft static pressure and the challenge of precisely defining anomalies through rules, traditional supervised models cannot be used in model selection. To address these issues, this paper proposes an empirical mode decomposition method combined with reconstruction error for shaft static pressure anomaly detection. The contribution of our method are as follows:

1. Proposed an anomaly monitoring method specifically for blast furnace shaft static pressure data. This method enables label-free anomaly detection while maintaining interpretable results that support further analysis incorporating process knowledge, transforming the current manual visual monitoring practice for anomaly detection.

2. Developed an innovative algorithmic framework combining improved Variational Mode Decomposition (VMD) with H-AAE network for anomaly detection. This framework specifically addresses the data characteristics of non-fixed-length periodicity in our dataset by optimizing VMD decomposition results through sample entropy and k-means clustering, achieving adaptive separation and reconstruction of trend, periodic, and detail variation components. These physically meaningful components are then integrated with the H-AAE network to enable simultaneous detection of different types of global and local anomalies.

3. Proposed a modified autoencoder H-AAE that incorporates concepts from Generative Adversarial Networks (GAN) and Huber loss function. This model ensures stability and computational efficiency by integrating GAN concepts into the autoencoder framework, while enhancing robustness in detecting both anomaly segments and points through the modified Huber loss function.

The rest of this document is organized as follows. Section 2 describes the details of the main process background and issues of the proposed method in this paper. Section 3 describes the main methodology proposed in this paper. Section 4 provides an industrial validation based on real data. Section 5 summarizes the work of this paper and its contributions.

## 2. Process Introduction and Problem Description

### 2.1. Blast Furnace Ironmaking Process

**Figure 1:**
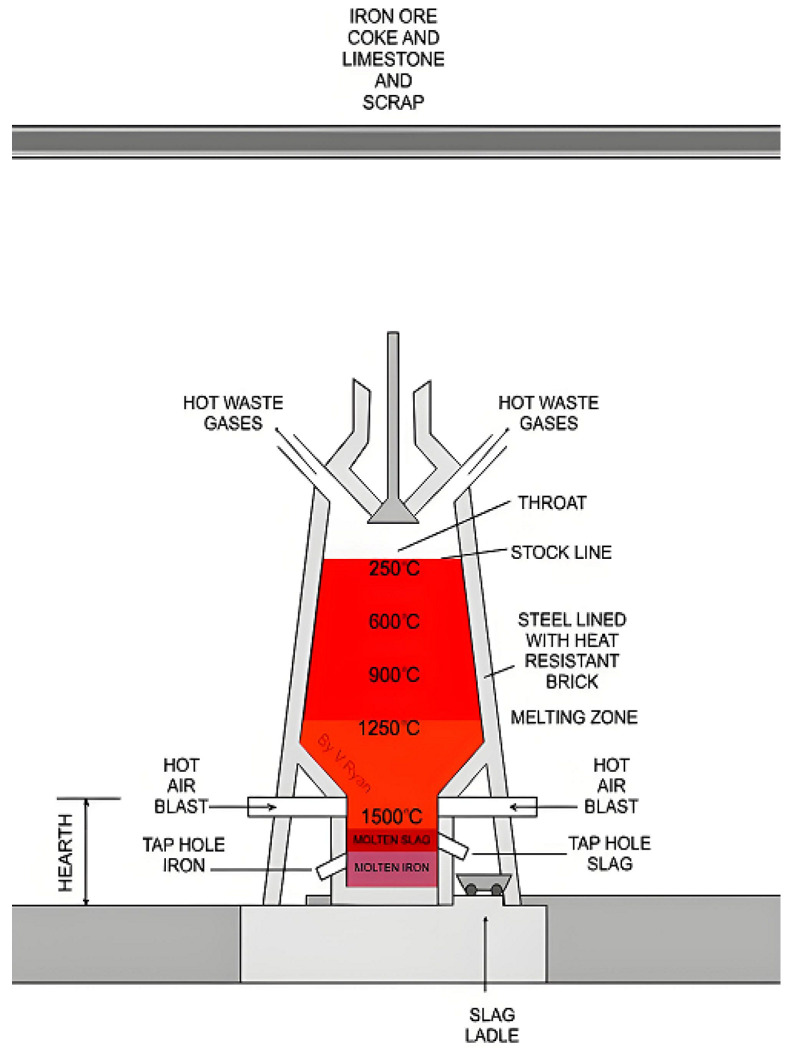
Main structure of blast furnace.

The production process of ironmaking focuses on the reduction in iron from ores and other natural forms of iron-containing compounds. The main methods of ironmaking include blast furnace, direct reduction and smelting. The basic principle is the reduction in ores to hot metal by physical and chemical reactions in a special atmosphere (reductants CO, H_2_, C, appropriate temperature, etc.). Most of the ore is used as raw material for ironmaking, except for a small portion of the ore. Blast furnace ironmaking is the main method of modern ironmaking and an important part of steel production. This method of ironmaking was developed and perfected on the basis of ancient open-hearth ironmaking. Although many new ironmaking methods have been researched and developed in different countries around the world, this method still accounts for more than 95% of the world’s iron production due to its high pressure furnace ironmaking technology, good economy, simple process, large output, high productivity and low energy consumption. In the ironmaking process, iron is made by loading raw materials (sintered ore, pelletized ore, iron ore), fuels (e.g., coke, pulverized coal), and other auxiliary materials (e.g., limestone, dolomite, manganese ore, etc.) into the furnace in a certain proportion to be smelted). Hot air is injected in certain proportions from the top of the blast furnace through the perimeter of the blast furnace to the bottom of the hot blast furnace, where they contribute to the combustion of the coke (depending on the blast furnace, pulverized coal, heavy oil, natural gas, and other auxiliary fuels may also be injected). At high temperatures, the coking coal is burned in the presence of oxygen in the air inside the drum. At high temperatures, the coking coal is burned by the oxygen in the blast furnace air, producing carbon monoxide and hydrogen. Raw materials, fuels plus smelting under the furnace and other processes, gas under the furnace and on the furnace discharge, heat transfer, reduction, smelting, decarburization effect and ore production in turn, adding slag iron ore raw materials to join and the combination of the flow in the furnace, the bottom of the furnace intermittent melting iron pots, sent to the steelmaking plant. At the same time, the production of blast furnace gas, blast furnace slag two kinds of by-products, the main non-ferrous metal impurities in the iron water, combined with limestone and other fluxes, from the output of the slag, all of which is used as a raw material for the production of cement, after water quenching; the production of the blast furnace gas produced by the outlet, after dust removal, into the hot blast furnace, blast furnace and coke oven, boiler, and other fuels. The main structure of the blast furnace is shown in the Figure 1.

### 2.2. Problem Description

During the production of the blast furnace, hot blast is injected through tuyeres, which includes fuels such as pulverized coal or natural gas. The hot air reacts with iron ore (mainly iron oxides), and the C in the blast material seizes the O in the iron ore, generating CO and CO_2_ that are discharged from the top of the blast furnace, and at the same time, the iron ore is reduced to hot metal. The hot air above forms a gas flow with reducing atmosphere in the blast furnace. Whether the blast furnace gas flow distribution is reasonably distributed, directly affects the reaction state of the blast furnace. Ensuring the reasonable distribution of airflow is the key to ensure the stable operation of the blast furnace.

Traditionally, the monitoring of blast furnace airflow status is mainly carried out through blast pressure, top pressure and other indicators for rough monitoring, and it is difficult to determine the overall airflow distribution in different locations of the blast furnace. In order to have a more detailed and comprehensive understanding of the blast furnace airflow distribution status, some steel mills in the blast furnace part of the stave coolers in multiple directions, circumferentially embedded embedded pressure sensors, to realize the different positions of the blast furnace radial gas flow profile status of real-time monitoring.

**Figure 2:**
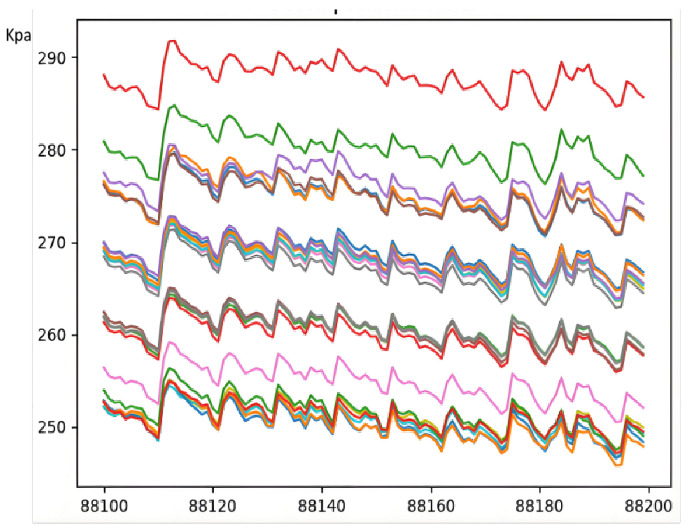
Furnace Static Pressure Data Fragment.

The operational stability of a blast furnace is closely tied to whether its shaft static pressure remains within normal ranges. For instance, certain anomalies in shaft static pressure may signal critical abnormalities like gas channeling within the furnace. Consequently, process experts must closely monitor changes in blast furnace shaft static pressure during operations (see Figure 2).

Due to the blast furnace there are burden charging, blast injection and other cyclical operations, such operations on the static pressure of the furnace at various points will have a cyclical impact, and due to changes in the production rhythm, errors, and other reasons, the length of such cycles is approximate but not the same. At the same time, for the judgment of airflow distribution anomalies in addition to each pressure measurement point itself is abnormal, multi-sensor between the relative value of the disorder is also one of the key anomalies that needs to be monitored.

Traditional anomaly detection methods commonly used in manufacturing, such as rule-based systems or simple degradation trend analysis, fail to meet the monitoring demands of the static pressure of the blast furnace shaft. Currently, no automated algorithms for identifying such anomalies are industrially deployed. Steel enterprises primarily rely on experienced operators to manually detect abnormalities through manual pattern recognition. However, as blast furnaces operate continuously 24/7, the intense workload of monitoring static pressure leads to frequent missed detections. This underscores the necessity to develop an automated monitoring algorithm tailored to the unique data characteristics of blast furnace static pressure.

Therefore, the rule-based anomaly recognition commonly used in the manufacturing industry is difficult to adapt to the monitoring scenario of the static pressure of the furnace, and the current iron and steel enterprises mainly recognize anomalies through the manual observation of experienced operators. Due to the 24 h uninterrupted production of the blast furnace, the intensity of the operator’s observation of the static pressure of the furnace is very high, and it is prone to omission of judgment and other problems. It is necessary to design a set of algorithms to adapt to the data characteristics of the static pressure of the furnace and realize automatic monitoring.

### 2.3. Problem Formulation

In this study, the shaft static pressure anomaly detection problem can be abstracted as a multidimensional time-series anomaly detection task. In time-series analysis, a single-variable time-series can typically be represented as a sequence of data points with temporal ordering:

(1) $$x=\{x_{1},x_{2},x_{3},\ldots ,x_{n}\}$$

where $x_{i}$ represents the data value at the *i*-th time point, and *n* is the total length of the time series.

The shaft static pressure data constitutes a multidimensional time-series variable. Assuming a blast furnace has *m* static pressure detection sensors labeled in positional order, the *j*-th time-series signal can be denoted as $x^{j}$, where j∈{1,2,…,m}.

Anomaly detection typically involves analyzing and making judgments on specific segments or windows of the time series. This study employs sliding window segmentation for detection segments. Assuming a window width of *t* and a step size of *k*, the *i*-th time segment can be represented as:

(2) $$W_{i}=\left[\begin{array}{cccc} x_{i\cdot k+1}^{1} & x_{i\cdot k+2}^{1} & \ldots & x_{i\cdot k+t}^{1}\\ x_{i\cdot k+1}^{2} & x_{i\cdot k+2}^{2} & \ldots & x_{i\cdot k+t}^{2}\\ \vdots & \vdots & \ddots & \vdots \\ x_{i\cdot k+1}^{m} & x_{i\cdot k+2}^{m} & \ldots & x_{i\cdot k+t}^{m} \end{array}\right]$$

where *i* is the window index and *k* is the step size (k∈{0,1,2,…}). The detection of anomalies in shaft static pressure essentially involves identifying anomalies in matrix $W_{i}$.

Since there are no labeled anomaly samples in the studied scenario, it is hard to evaluate the model using metrics like accuracy and recall from classification tasks.This paper adopts the idea of unsupervised algorithms, defining that the anomaly degree of a segment $W_{i}$ increases with the distance ($W_{i},W_{c}$) from the normal state $W_{c}$ (represented by most segments). Note that $W_{c}$ is not necessarily a matrix but represents the concept of the normal state.A method will be designed to calculate the distance ($W_{i},W_{c}$) for the static pressure scenario.

## 3. Method

This section is divided into three parts: Anomaly Detection Method, Time-Series Data Processing, and implementation details.

### 3.1. Anomaly Detection Method

For the normal state representation $W_{c}$, this study adopts a reconstruction error-based approach. The encoder of the Autoencoder (AE) maps input $W_{i}$ to latent variables *Z*, while the decoder reconstructs the input space through *R*. Since the primary task in this scenario involves detecting temporal segment anomalies, segment-level anomalies may manifest as various patterns such as trend shifts, fluctuations, or occasional extreme values. The L2 loss is highly sensitive to extreme values, which could lead the model to become less sensitive to other types of anomalies.To enhance robustness against outliers, the original L2 loss is replaced with the Huber loss, whose sensitivity to anomalies can be controlled via parameter δ. The AE is trained by minimizing the Huber loss between inputs and reconstructions:

(3) $$\min\left\{H_{\delta }\left(W_{i},\mathrm{AE}\left(W_{i}\right)\right)\right\}$$

where the Huber loss is defined as:

(4) $$H_{\delta }\left(a,b\right)=\left\{\begin{array}{cc} \frac{1}{2}{\left(a-b\right)}^{2} & \mathrm{if}\;|a-b|\leq \delta \\ \delta |a-b|-\frac{1}{2}\delta^{2} & \mathrm{otherwise} \end{array}\right.$$

To balance computational efficiency and industrial applicability, the adversarial training architecture from [26] is integrated with the AE. This hybrid framework consists of an encoder *E* and two decoders $D_{1}$, $D_{2}$. The core adversarial objectives are reformulated using Huber loss:

(5) $$\left\{\begin{array}{c} \min_{{\mathrm{AE}}_{1}}\left\{H_{\delta_{1}}\left(W_{i},{\mathrm{AE}}_{2}\left({\mathrm{AE}}_{1}\left(W_{i}\right)\right)\right)\right\}\\ \max_{{\mathrm{AE}}_{2}}\left\{H_{\delta_{2}}\left(W_{i},{\mathrm{AE}}_{2}\left({\mathrm{AE}}_{1}\left(W_{i}\right)\right)\right)\right\} \end{array}\right.$$

The anomaly scoring function is accordingly modified to:

(6) $$A\left(\hat{W_{i}}\right)=\alpha H_{\delta_{3}}\left(\hat{W_{i}},{\mathrm{AE}}_{1}\left(\hat{W_{i}}\right)\right)+\beta H_{\delta_{4}}\left(\hat{W_{i}},{\mathrm{AE}}_{2}\left({\mathrm{AE}}_{1}\left(\hat{W_{i}}\right)\right)\right)$$

with α+β=1, where $\delta_{1}$–$\delta_{4}$ are tunable hyperparameters optimized through cross-validation for specific industrial environments. This design mitigates the oversensitivity of L2 loss to outliers while preserving its stability in smooth regions.

Since this study uses a reconstruction error-based anomaly detection approach and assumes the training set is approximately normal or contains minimal anomalies, the anomaly threshold is set as the 99th percentile of the training set scores:

(7) $$T=Q_{0.99}\left(A(\hat{W})\right)=\inf\left\{x\in R:P\left(A(\hat{W})\leq x\right)\geq 0.99\right\}$$

### 3.2. Time Series Data Processing

While the adversarial training architecture described above can construct $W_{c}$, its optimization objective focuses on minimizing the overall Huber difference in the data. However, the shaft static pressure data contains multiple superimposed periodicities with variable lengths. Solely considering overall error tends to overlook detailed anomalies, which affects model performance. To address this data characteristic, the time-series segments used for anomaly detection are decomposed.

Variational Mode Decomposition (VMD) is a non-recursive signal processing method that decomposes time-series data into a series of Intrinsic Mode Functions (IMFs) with finite bandwidth. Compared to traditional Empirical Mode Decomposition (EMD), VMD obtains IMF components through iterative optimization, effectively avoiding mode mixing and producing more accurate and stable decomposition results. The VMD objective function can be expressed as:

(8) $$\left\{\begin{array}{c} \min\left\{\sum_{k=1}^{K}{∥\partial_{t}\left[\left(\delta \left(t\right)+\frac{j}{\pi t}\right)*u_{k}\left(t\right)\right]e^{-j\omega_{k}t}∥}_{2}^{2}\right\}\\ s.t.\sum_{k=1}^{K}u_{k}=f\left(t\right) \end{array}\right.$$

where $\partial_{t}$ denotes the partial derivative, δ(t) is the Dirac delta function, “*” represents the convolution operation, *K* is the total number of components, and f(t) is the original signal.

For the decomposed signal components, process experts focus on overall trend changes and short-term detailed variations. From the perspective of visual inspection aligned with process analysis logic, some components represent overall trends while others represent detailed changes. In terms of information content, these two types of components exhibit significant differences, which can be distinguished by calculating sample entropy that characterizes time-series complexity. Therefore, this study designs a method combining sample entropy with k-means clustering to extract components representing overall trends and detailed variations from the VMD decomposition results and classify them accordingly.

The calculation logic is as follows:

1. Given a time series: {x(i),i=1,2,…,n}.
2. Sequence segmentation: Divide the time series into k=n−m+1 vectors using a window length *m*. Each vector sequence is represented as: $X_{i}\left(t\right)=\left\{x_{i}\left(t\right),\;x_{i+1}\left(t\right),\;\ldots ,\;x_{i+m-1}\left(t\right)\right\}$.
3. Distance calculation: Compute the distance between each *m*-dimensional vector sequence and all other *k m*-dimensional vector sequences. The distance is defined as the maximum absolute difference between corresponding elements of two vectors:
  (9) $$d_{ij}=\max\{|x_{i+k}\left(t\right)-x_{j+k}\left(t\right)\left|\right\},\;k=0,1,...,m-1$$
4. Threshold definition: F=r·SD, where *r* is a coefficient (typically 0.1–0.25) and SD is the standard deviation of the sequence.
5. Ratio statistics: Count the ratio of *m*-dimensional vector sequences with distances exceeding *F* to the total number (excluding self-comparisons), denoted as $C_{m}\left(i\right)$. Calculate the average of all $C_{m}\left(i\right)$, denoted as ${⌀}_{m}\left(t\right)$.
6. Sample entropy calculation: Repeat steps 2–5 with window length m+1 to obtain ${⌀}_{m+1}\left(t\right)$. Compute sample entropy using:
  (10) $$\mathrm{SampEn}\left(t\right)=\ln{⌀}_{m}\left(t\right)-\ln{⌀}_{m+1}\left(t\right)$$
7. Sample entropy clustering: Cluster the VMD-decomposed sequences using k-means into three categories representing trend, cycle, and residual fluctuations. Combine components within each cluster to form three final components: $W_{i}^{1}$ (trend), $W_{i}^{2}$ (cycle), and $W_{i}^{3}$ (residual).

Using these three components as input, the adversarial training-based reconstruction error minimization method is applied for anomaly detection. Three anomaly scores are obtained: $A(\hat{W_{i}^{1}})$, $A(\hat{W_{i}^{2}})$, and $A(\hat{W_{i}^{3}})$. The final anomaly detection results are the union of anomalies detected from all three components.

### 3.3. Implementation Framework

**Figure 3:**
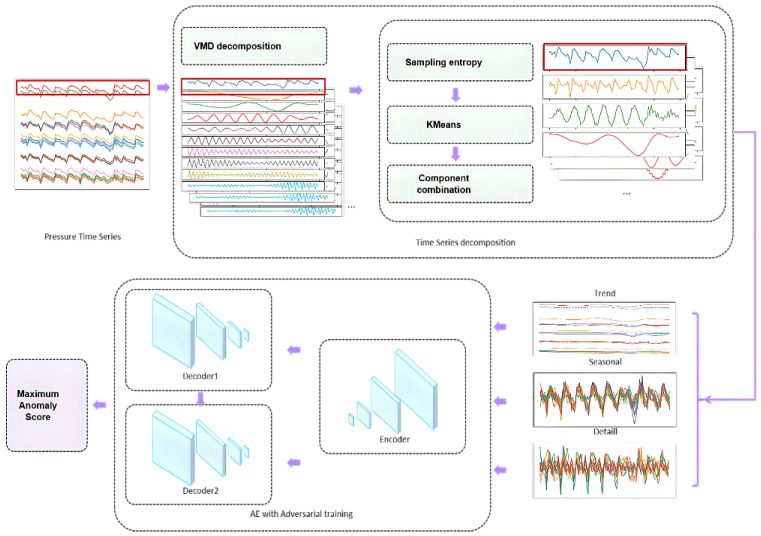
Workflow of the anomaly detection algorithm.

The complete implementation workflow of the proposed unsupervised anomaly detection algorithm for blast furnace multidimensional shaft static pressure data is shown in Figure 3.

The algorithm steps are described as follows:

1. **Step 1**: Perform VMD decomposition on the raw data to obtain multiple components. Since subsequent steps involve post-processing the VMD decomposition results, this paper does not focus on adaptive parameter adjustment for VMD. The raw data are preliminarily split into 10 components, retaining dynamic baseline drift, with the convergence criterion set to “$10^{-7}$”.
2. **Step 2**: Due to the variable-length periodic characteristics of the data and the need for model interpretability, post-processing of the IMF components decomposed in Step 1 is required. Key steps include calculating the sample entropy of different components, standardizing the data to eliminate dimensional effects, and setting the tolerance to 0.1 to balance robustness and noise impact. Further, k-means clustering is applied to the entropy values, categorizing the components into three classes—trend, periodic, and residual (remaining details)—based on data morphology. Thus, the number of clusters is set to 3. The clustered components are recombined to decompose the original time series data into trend, periodic, and residual components. This separation mitigates the influence of periodic fluctuations and impact-type anomalies on model performance.
3. **Step 3**: Since the shaft static pressure data is multi-dimensional time series, after decomposing each time series via VMD and regrouping them into three components, identical component types across multiple dimensions are grouped to form three 2D arrays. These arrays are individually input into the improved H-AAE network for training and anomaly detection. Given the presence of multiple anomaly types in this scenario, each with distinct physical meanings and operational implications, the detected anomaly results from each component in the H-AAE model are combined via a union operation. All anomalies are presented to process experts, who evaluate the necessity and approach for handling them by integrating other production parameters.

## 4. Experiments

### 4.1. Dataset Description

Due to frequent changes in operating conditions during steel production that lead to data distribution shifts, high-frequency model retraining using recent data is typically required in actual production. To validate the model’s effectiveness, this study selects time segments containing shaft static pressure anomalies and their preceding periods for training and testing, effectively simulating real production conditions.

The experimental dataset consists of real minute-level shaft static pressure data collected from multiple sensors at a steel plant, totaling 7500 min. The first 3500 min are designated as the training set (assumed to contain almost no anomalies), and the remaining 4000 min as the test set.

### 4.2. Evaluation Methodology

Time-series segments are obtained through sliding window partitioning with specified window widths. In this dataset, anomalies are manually labeled by process experts at the window level. Windows containing anomalies are labeled as 1, and normal windows as 0.

The primary evaluation metrics are precision, recall, and F1-score:

(11) $$\mathrm{Precision}=\frac{TP}{TP+FP}$$

(12) $$\mathrm{Recall}=\frac{TP}{TP+FN}$$

(13) $$F_{1}-\mathrm{score}=\frac{2\cdot \mathrm{Precision}\cdot \mathrm{Recall}}{\mathrm{Precision}+\mathrm{Recall}}$$

where TP represents true positives, FP false positives, and FN false negatives.

### 4.3. Experimental Results

The shaft static pressure data exhibits periodicity and certain trend characteristics. Whether providing richer data information for anomaly state detection or supplying fundamental analysis data (such as long-term furnace profile trends and short-term local gas flow analysis) for process experts through decomposition, developing an algorithm that correctly decomposes trend, cycle, and residual components is crucial.

#### 4.3.1. Comparison Experimental Results

**Figure 4:**
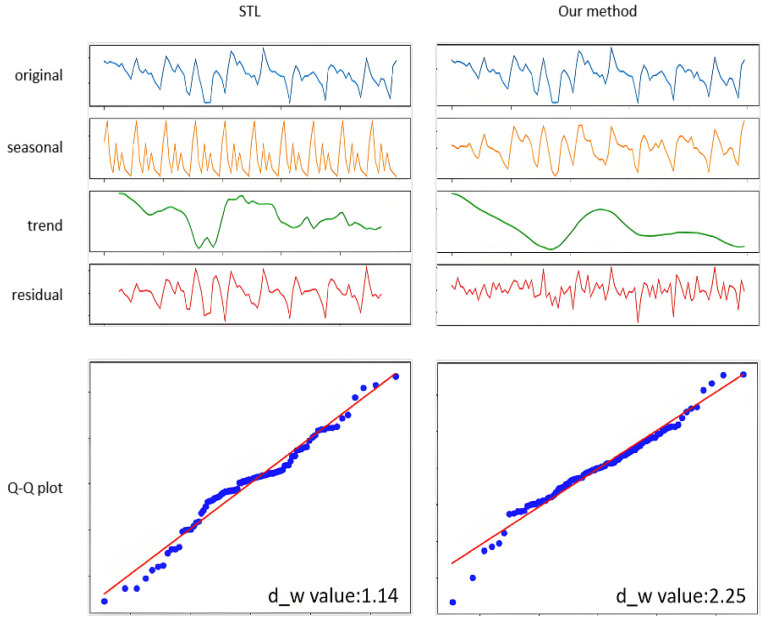
Comparison of decomposition effects (**Left**: STL, **Right**: Proposed Method).

Since the periodic fluctuations in shaft static pressure are related to the burden distribution cycle, traditional approaches typically perform decomposition using STL method [34,35] with a fixed 10-min cycle. However, due to variable gas flow patterns inside the blast furnace, the actual period length and morphology of shaft static pressure are inconsistent. Therefore, this paper proposes a VMD decomposition method combined with sample entropy and clustering algorithms. The decomposition effects are compared in Figure 4.

Key observations from Figure 4 include:

- The upper part of Figure 4 illustrates the contrasting extraction effects of STL and the proposed method on trend, periodic, and residual components. By observing the morphology of each component in the time-series plots, it is evident that the original data exhibits oscillatory periodic fluctuations with variations in cycle length and morphological details, alongside a gradual declining trend. The proposed method achieves accurate extraction of the actual periodic patterns while successfully isolating significant trend components, ensuring the extracted features maintain physically interpretable characteristics. In contrast, STL decomposition requires predefined cycle lengths, leading to extracted periodic components that deviate markedly from observed patterns. This suboptimal periodic extraction further causes incomplete separation of trend and residual components from cyclic influences. Notably, residual components fail to reliably identify impact-type anomalies due to uncorrected periodic interference.
- The lower part of Figure 4 further analyzes the decomposed residual components using Q–Q plots. Results indicate that residuals from our method show no significant autocorrelation (Durbin–Watson statistic between 1.5 and 2.5), with most scatter points clustered near and parallel to the red reference line. In contrast, STL residuals demonstrate moderate positive autocorrelation (DW = 1.14), exhibiting oscillatory scatter patterns around the red line. The Q–Q plot also reveals that extreme residual values correspond to localized anomalies.

This paper conducts a comparison between its proposed model and various classical unsupervised anomaly detection models. In this paper, four unsupervised classical models supporting multi-dimensional anomaly recognition, namely IsoForest, LOF, PCA, and HBOS, are selected for comparison. Since the method of this paper mainly recognizes segment anomalies, for full comparison, the above classical models are used to capture the anomalies firstly, and then the sliding window is cut according to 60 window widths and 1 as the step size, and the proportion of anomalies in the sliding window is higher than 20%, then the segment is considered to be anomalous. The results of the comparison experiments are shown in the following Table 1.

**Figure 5:**
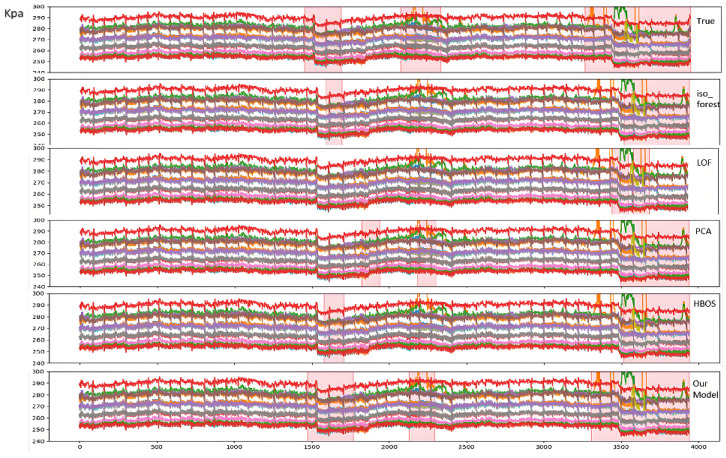
Anomaly detection comparison (Ground Truth vs. Algorithm Results).

The test fragment specific labeled anomalies are plotted against the anomalies recognized by the different algorithms as follows:

The pink semi-transparent box in the above figure indicates the abnormality segments identified by manual marking or algorithm. As this paper intercepts the time series data fragments for anomaly identification by means of sliding window, the window width is temporarily set at 60, so the starting point of the anomaly fragments labeled in the figure is earlier than the actual anomaly moment, but it does not affect the effect of real-time on-line alarms in actual production, and it does not affect the effect of real-time on-line alarms in actual production. The Figure 5 reveals that process experts have flagged anomalies worth attention, including abnormally high/low values, sudden drops/increases, significant trend shifts, and abnormal fluctuations. Among the classic anomaly detection algorithms selected for comparison, only PCA identifies some notable trend variations, while others primarily detect extreme value outliers. Quantitative analysis shows recall rates of 0.46, 0.19, 0.50, and 0.49 for Isolation Forest, LDF, PCA, and HBOS, respectively, with the highest recall being merely 0.5, indicating severe under-detection issues in traditional methods. The proposed algorithm demonstrates superior accuracy and recall, effectively capturing diverse anomalies like numerical deviations, fluctuation patterns, and abrupt changes. Subsequent ablation experiments will clarify which modules significantly contribute to recall improvement.

#### 4.3.2. Ablation Experimental Results

Further ablation experiments comparing different models are presented in Table 2. The proposed method achieves 0.93 F1-score, outperforming baseline models.

**Figure 6:**
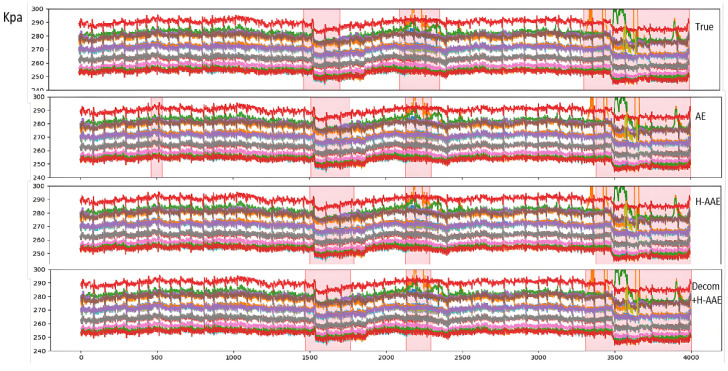
Anomaly detection comparison (Ground Truth vs. Algorithm Results).

Figure 6 compares manual annotations with algorithmic detection results. The pink translucent boxes represent human expert annotations, while blue boxes show algorithm detections.

**Figure 7:**
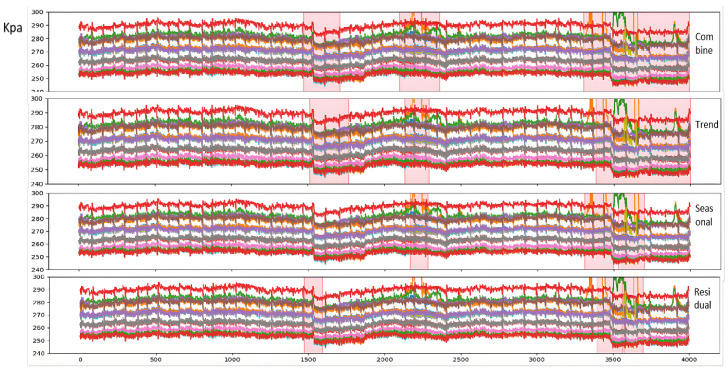
Anomaly detection by component (Trend/Cycle/Residual).

Notably, while most models can detect significant trend anomalies (e.g., sustained high/low values), our method better captures morphological changes and sudden transitions that align with manual expert judgments. Further analysis of detection results from different components is shown in Figure 7.

- **Anomaly Segment Annotation**: The pink semi-transparent boxes in the figure indicate anomaly segments manually labeled or algorithmically identified. Due to the sliding window approach (window width = 60) used in this study for extracting time-series data fragments, the annotated anomaly start points precede actual anomaly timestamps. This design does not compromise the real-time online alarm performance in practical production.
- **Model Comparison**: Observations from Figure 6 reveal that while both AE and H-AAE models can identify anomaly segments overlapping with manual annotations, the AE model only detects anomalies characterized by “significant sustained elevation or reduction” in specific segments. For example:The second anomaly segment identified by AE corresponds to a sharp global decline followed by gradual recovery; The third AE-detected anomaly segment reflects a distinct “bulge” morphology in the data; The fourth AE-detected segment combines both “bulge” and sharp decline anomalies; Additionally, AE erroneously flags the first anomaly segment due to poor robustness.
  To address AE’s robustness limitations, this study integrates GAN mechanisms and modifies the loss function, enabling H-AAE to recognize broader anomaly types. The modified H-AAE avoids AE’s false identifications (e.g., Segment 1) but remains reliant on detecting “significant sustained variations”, showing only marginal metric improvements.
  After introducing VMD postprocessing to decompose trend, periodic, and residual components, the model demonstrates enhanced performance: our model identifies anomalies during early trend declines (e.g., Anomaly segment 1 corresponding to AE’s Segment 1 and H-AAE’s Segment 2), demonstrating short-term trend detection capability; e.g., Anomaly segment 3 (mapped to AE’s Segment 4 and H-AAE’s Segment 3) successfully captures short-term fluctuations; quantitative metrics show substantial improvements in accuracy and recall, confirming the critical role of signal decomposition.
- **Component-Wise Anomaly Analysis**: Trend Component: Detects significant long-term increases/decreases. Undecomposed data risks masking other anomaly types under dominant trends; Periodic Component: Improved via Huber-modified loss functions, effectively identifying transient or sustained “fluctuation” patterns; Residual Component: Captures spike anomalies and short-term deviations, enabling early micro-anomaly warnings. Final anomaly results combine outputs from all three components. Component-specific anomaly types aid fault diagnosis and severity assessment, enhancing interpretability; Fusion logic adapts dynamically to operational requirements, enabling refined detection and alert strategies.
  While most models detect extreme-value anomalies, our method excels in identifying subtle anomalies (e.g., morphological shifts, abrupt changes) akin to expert judgment. The trend, periodic, and residual components, respectively, specialize in global trends, cyclical fluctuations, and transient peaks, with flexible fusion strategies supporting customized industrial needs.

## 5. Conclusions

This paper conducts an in-depth investigation into the anomaly detection problem in static pressure data. Given the scarcity of anomaly samples and the lack of labeled datasets, we propose an unsupervised anomaly detection approach based on reconstruction errors. First, to address the issues of traditional autoencoder (AE) models being sensitive to outliers and having poor stability, we introduce the Huber loss function into the AE framework to mitigate the sensitivity of L2 loss to outliers, and integrate a dual-decoder generative adversarial network (GAN) to enhance model stability through adversarial training. Second, to tackle the challenges posed by the variable-length periodic patterns in shaft static pressure data, where anomaly detection based on raw data often fails to capture multi-category anomalies such as trend deviations and periodic disruptions, we employ Variational Mode Decomposition (VMD) to decompose the original signals. This decomposition is further optimized through sample entropy analysis and clustering, adaptively separating the signals into trend, periodic, and residual components while ensuring residual normality and eliminating autocorrelation. By feeding these decomposed components into the adversarially enhanced AE model, our method achieves multi-dimensional detection of long-term trend shifts, periodic pattern disruptions, and transient fluctuations. Comparative and ablation experiments validate the superiority of the hybrid framework. Furthermore, the interpretable component-level results derived from this decomposition enable operators to perform detailed analysis of different anomaly types and implement targeted adjustment measures.

## Figures and Tables

**Table 1:** Performance comparison of different models.

Model	Precision	Recall	F1-Score
IsoForest	1.00	0.46	0.63
LOF	1.00	0.19	0.31
PCA	0.82	0.50	0.62
HBOS	1.00	0.49	0.66
Proposed	0.95	0.91	0.93

**Table 2:** Performance comparison of different models.

Model	Precision	Recall	F1-Score
AE	0.88	0.82	0.85
H-AAE	0.92	0.82	0.86
Proposed	0.95	0.91	0.93

## Data Availability

Where data is unavailable due to privacy restrictions.
